# Insight and Associated Factors among Patients with Schizophrenia in Mental Specialized Hospital, Ethiopia, 2018

**DOI:** 10.1155/2019/2453862

**Published:** 2019-11-17

**Authors:** Mandaras Tariku, Demeke Demilew, Tolesa Fanta, Meskerem Mekonnen, Dessie Abebaw Angaw

**Affiliations:** ^1^Department of Psychiatry, College of Health and Medical Science, Haramaya University, Harar, Ethiopia; ^2^Department of Psychiatry, College of Medicine and Health Science, University of Gondar, Gondar, Ethiopia; ^3^Amanuel Specialized Mental Hospital, Addis Ababa, Ethiopia; ^4^Department of Epidemiology and Biostatistics, Institute of Public Health, College of Medicine and Health Science, University of Gondar, Gondar, Ethiopia

## Abstract

**Background:**

Insight is the degree of the patient's awareness and understanding of their attributions, feelings, behavior and disturbing symptoms. Majority of the patients with schizophrenia have poor insight and insight is an important prognostic indicator in schizophrenia to enhance treatment compliances and reducing the risks of clinical deterioration. The main objective of this study was to assess insight and its associated factors among patients with schizophrenia at mental specialized hospital in Ethiopia.

**Methods:**

Institutional based cross-sectional study was conducted from May to June 2018 Mental Specialized Hospital among 455 patients with schizophrenia. Insight was measured by an abridged version of Scale to assess unawareness of mental disorder. Positive and Negative Syndrome Scale, Calgary depressive scale, Oslo social support scale was used to identify factors associated with insight. Simple and multiple linear regression analysis were used to assess associated factors of insight in the participants.

**Results:**

The mean score of insight was 13.66 (95% CI 13.27, 14.04). Age at first onset of illness, duration of treatments, depressive symptoms were inversely associated with mean insight score; whereas unemployed, positive and negative syndrome, previous hospitalization, >=2 episodes were positively associated with mean insight score.

**Conclusion:**

Nearly half of the study participants were scored above the mean insight score so, the clinicians and psychotherapists should have to work together to improve insight among patients with schizophrenia.

## 1. Background

Insight in mental health is the degree of the patient's awareness and understanding of their attributions, feelings, behavior and disturbing symptoms; self-understanding as well as the potential causes of psychiatric presentation [1].

Insight is viewed as multidimensional variable of the realization of the need for treatments, ability to re-label unusual mental events as pathological and attribute appropriate causes for mental illness. Insight assessment is an important aspect of diagnosis, treatments and prognosis of the patient with mental illness [2–4].

Unawareness of having mental illness among the patients with schizophrenia viewed as an independent phenomenon rather than the secondary manifestation of schizophrenia symptom [5, 6].

WHO international pilot study showed that 98% patients with schizophrenia had a lack of insight and systematic study revealed that between 50% and 80% of patients with schizophrenia had characterized by poor insight [3, 7, 8].

The consequences of lack of insight are failure to recognize the need for treatment; leading to medication nonadherence and impacts on the patients, families, communities and affects the course of illness [9, 10].

Insight in the patient with schizophrenia has been associated with the depressive symptoms, positive and negative syndromes of schizophrenia, unemployed, relapse and rehospitalization [11–13].

Female and depressive symptoms are associated with good insight whereas symptoms severity, positive and negative syndrome are associated with poor insight [13, 14].

Although insight assessment is a crucial aspect of patients with schizophrenia there are few published studies in Africa and no published study in Ethiopia. Therefore, the main aim of this study was assessing insight and its associated factors among patients with schizophrenia at mental specialized hospital in Ethiopia.

## 2. Methods

### 2.1. Study Setting and Participants

An institutional based cross-sectional study was conducted from May to June 2018 G.C at Amanuel Mental Specialized Hospital located in Addis Ababa. The hospital provides care for patients coming from the entire nation of Ethiopia. Among 3420 patients with schizophrenia who had regular follow up at AMSH; 455 were recruited for the study and those acutely ill and patients with intellectual disability who had schizophrenia were excluded from the study. The samples were selected through systematic random sampling with the interval of 8.

### 2.2. Instruments

Structured clinical interview for DSM-4 (SCID) was used to confirm a diagnosis of schizophrenia. Insight was measured through interviews by using an abridged version of scale to assess unawareness of mental disorder (SUMD). It has 9 items self-report measure: awareness of the mental disorder, awareness of achieved effect of medication, awareness of social consequence of mental disorder and awareness of recognition of symptoms rated as 0 (not indicated), 1 (aware), 2 (somewhat aware/unaware ), 3 (severely unaware) and the Cronbach's alpha was 0.89 and the high scores indicates poor insight [15]. *Positive syndrome: *based on positive syndrome scale of PANSS (7–49) higher scores indicate the presence and severity of positive syndrome, *Negative syndrome: *based on Negative syndrome scale of PANSS (7–49) higher scores indicates the presence and severity of negative syndrome, *Depressive symptoms: *patients with schizophrenia scoring >6 using Calgary depression scale for schizophrenia, *Social support: *based on Oslo social support scale scores between (3–8) are poor social support, (9–11) moderate social support and (12–14) strong social support [16–20].

### 2.3. Data Processing and Analysis

Data were checked for completeness and consistency and entered into Epidata version 3.1 and analyzed by using statistical package software for social sciences (SPSS) version 20. Simple and multiple linear regression analysis were carried out to explain the association between explanatory and outcome variables. The strength of association was measured by *β* coefficient with 95% confidence interval and the Level of significance was declared at the *p*-value of <0.05.

## 3. Results

### 3.1. General Description of the Study Participants

A total of 445 patients responded to the questionnaire, which yields a response rate of 97.8%. The mean age of the respondents was 36.1 ± 47.8 years. Majority of the participants were male and 164 (36.9%) were unemployed (Table 1).

### 3.2. Clinical Characteristic of the Study Participants

Among the total of 445 participants, 259 (58.2%) had more than two episodes of schizophrenia and 240 (53.9%) had previous history of hospitalization. The mean age of the first onset of schizophrenia was 25.7 ± 5.2 years and the mean duration of treatment was 6.64 ± 3.51 years. The mean of the positive and negative syndromes were 22.67 ± 6.12 and 22.36 ± 3.69 respectively and 111 (24.9%) had depressive symptoms and majority of the participants were nonadherent to the medication (Table 2).

### 3.3. Social Support Characteristics of Study Participants

Among the total of the study participants, 264 (59.3%) had poor social support, 74 (16.6%) had moderate social support and 107 (24%) had good social support.

### 3.4. Scale to Assess Unawareness of Mental Disorder (SUMD)

The mean total score of insight was 13.66 ± 4.21 (95% CI 13.27, 14.04) as measured in a range from 3 to 27 using SUMD an abridged version and (49.2%) of the participants were scored above the mean total insight score.

Based on multivariate analysis 88.1% of the variation of insight was explained by the variables in the model. The mean insight score of study participants who were unemployed was increased by 1.50 units (95%CI 1.09, 1.92) as compared with those who were government employed and the study participants who had more than two episodes had their mean insight score increased by 0.95 units (95%CI 0.61, 1.29) as compared with those who had the first episode (Table 3).

## 4. Discussion

The mean total score of insight among patients with schizophrenia was 13.66 ± 4.21 (95% CI 13.27, 14.04) and 49.2% of the participants were scored above the mean score. The result was supported by a cross-sectional study in Peru (13.28 ± 8.678) [21], Taiwan (13.1 ± 7.4) [22] and Netherlands (13.6 ± 6.7) [23]. The current finding was higher than a cross-sectional study conducted in Malawi (6.8 ± 4.1) [24], Ghana (8.32 ± 6.22) [25] and China (9.7 ± 5.6) [26] which showed that the total mean insight score of the participants were low. The possible reasons could be sample size difference (60 in Malawi, 49 in Ghana, 139 in china) and measurement difference in assessing insight (Schedule for assessment of insight in both Malawi and Ghana study whereas, Insight and treatment attitude questionnaires in China. The result also not supported by a cross-sectional study in Nigeria (9.6 ± 4.7) [27] by using SUMD. This discrepancy could be justified as the study participants were low mean scores of psychopathology (the mean positive and negative syndrome was 13.08 and 19.92 respectively), sample size differences (312 samples), excludes new patients and participants' take medication for more than 1 year included in the Nigeria study.

The current study revealed that being unemployed was positively associated with the mean total insight score. The result was in agreement with a comparative study conducted in Italy [28]. The possible reasons could be aberrant positive symptoms of schizophrenia (hallucination or other symptoms of psychosis) and prefrontal dysfunction which affect neural transmission in Central nervous system that could altered working memory.

According to this study; having more than two episodes and previous hospitalizations were positively associated with mean total insight score. The findings were consistent with different studies in Bulgaria [29], Taiwan [22] and New York [12], stated that more than two episodes and previous hospitalization were significantly associated with poor insight. The results could be justified as the patient with schizophrenia has biopsychosocial causality, the nature of the illness, poor adherence to medication and poor social support systems.

This study also showed that age at first onset of illness and duration of treatments was inversely associated with the mean total insight score. Supported by different studies conducted in Canada [30], Italy [28], London [31], and USA [32] which showed that the older age of first onset and the long-term duration of treatments predicted good insight. The possible explanation of improvement of insight with long term duration of treatments is the effect of medication and the patient spends more time in a medical setting and hearing psychiatric terminology and they may tend to pick up relevant words as the result of learning effect. There might be increasing social interaction and health information seeking behaviors among older age that would be likely to decreases mean insight score.

The positive and negative syndromes of schizophrenia were positively associated with the mean insight score in this study. The findings were consistent with the different studies in Ghana [25], Canada [33], London [31] and USA [34] That showed the positive and negative syndromes of schizophrenia predicting poor insight. The possible reasons could be that positive and negative syndromes of schizophrenia are a result of a deficit in frontal cortex and cortical midline region of the brain that could also be the reasons for insight processing [35, 36].

The current study revealed that presence of depressive symptoms was negatively associated with mean total insight score. The finding was in agreement with the different studies in Ghana [25], Canada [33], Italy [37] and New York [12] stated that patients with more insight have depressive symptoms. The reason could be justified as the patient with schizophrenia has been characterized by multiple domains of symptoms. Depressive symptoms are most commonly presented at residual phase after controlling active symptoms of the schizophrenia. The patients are likely to start to regain their awareness toward mental illness and consequence of mental illness; as a result, they could internalize the situation and develop depression. This result was also in further support of “insight paradox” (describes the presence of depressive symptoms among patients with schizophrenia who have a good level of insight).

## 5. Conclusion

This study revealed that the mean total insight score was moderate. Unemployed, having more than two episodes, previous hospitalization, positive syndrome, negative syndrome, and medication nonadherence were positively associated with the mean total insight score whereas age at onset of illness, duration of treatments and presence of depressive symptoms were inversely associated with the mean total insight score among patients with schizophrenia.

## 6. Limitation of the Study

The limitation of this study might be that some variables are affected by recall bias and that the scale to assess unawareness of mental disorder is not validated in Ethiopia.

## Figures and Tables

**Table 1:** Description of socio-demographic characteristics of patients with schizophrenia.

Variables	Category	Frequency	Percentage
Sex	Female	170	38.2
	Male	275	61.8

Religion	Orthodox	211	47.4
	Muslim	179	40.2
	Others^∗^	55	12.4

Marital status	Single	222	49.9
	Married	144	32.4
	Separated	45	10.1
	Others^∗∗^	34	7.6

Ethnicity	Amhara	170	38.2
	Oromo	131	29.4
	Gurage	91	20.4
	Others^∗∗∗^	53	11.9

Educational status	Unable to read and write	142	31.9
	Able to read and write	204	45.8
	Primary school	43	9.7
	High school	24	5.4
	College graduate and above	32	7.2

Occupation	Government employed	88	19.8
	Merchant	47	10.6
	Farmer	47	10.6
	Student	49	11
	Day laborer	29	6.5
	House wife	21	4.7
	Unemployed	164	36.9

Others^∗^; catholic, protestant others^∗∗^; widowed, divorced others^∗∗∗^; Tigre, Silte, Woleyta.

**Table 2:** Description of clinical characteristic of patients with schizophrenia.

Variables	Category	Frequency	Percentage (%)
Number of episodes	First episode	186	41.8
	≥2 episodes	259	58.2
Previous hospitalization	Yes	240	53.9
	No	205	46.1
Depressive symptoms	Yes	111	24.9
	No	334	75.1
Age at first onset of illness mean (SD)		25.70 (5.22)	
Duration of treatment mean (SD)		6.64 (3.51)	
Positive syndrome mean (SD)		22.67 (6.12)	
Negative syndrome mean (SD)		22.36 (3.69)	
General psychopathology mean (SD)		27.42 (10.45)	
PANSS total mean (SD)		72.44 (14.00)	

**Table 3:** Factors associated with insight among patients with schizophrenia at mental specialized hospital, Addis Ababa, Ethiopia June, 2018.

Variables	*β* coefficient (95% CI)
Male	0.15 (−1.50, 0.44)
Female	0
Unemployed	1.50 (1.09, 1.92)^∗∗^
Government employed	0
Unable to read and write	0.21 (−0.09, 0.51)
College graduate and above	0
Age at first onset of illness	−0.15 (−0.20, −0.11)^∗∗^
Duration of treatments	−0.06 (−0.11, −0.01)^∗^
More than two episodes	0.95 (0.61, 1.29)^∗∗^
First episode	0
Previous hospitalization	1.61 (1.18, 2.04)^∗∗^
No previous hospitalization	0
Positive syndrome	0.10 (0.06, 0.33)^∗∗^
Negative syndrome	0.50 (0.10, 0.20)^∗∗∗^
Depressive symptoms	−2.50 (−3.03, −2.05)^∗∗^
No depressive symptoms	0
Poor social support	−0.05 (−0.57, 0.48)
Moderate social support	−0.11 (−0.62, 0.40)
Strong social support	0

Note: *VIF*_max_ = 3.51, constant = 11.00 (95%CI 9.21, 12.77). Model summary: *R*^2^ = 88%, adjusted *R*^2^ = 87.6%, and *F* (12, 432) = 263.14 with *p* value <0.001. ^∗∗^ = statistically significant (*p* value <0.001) and ^∗^ = statistically significant (*p* value <0.05).

## Data Availability

The data sets used and/or analyzed during the current study are available from the corresponding authors on reasonable request.

## Abbreviations

AMSH: Amanuel mental specialized hospital
PANSS: Positive and negative syndrome scale
SD: Standard deviation
SPSS: Statistical package of social science
SUMD: Scale to assess unawareness of mental disorder
WHO: World health organization.
